# Speculations on biting midges and other bloodsucking arthropods as alternative vectors of *Leishmania*

**DOI:** 10.1186/1756-3305-7-222

**Published:** 2014-05-14

**Authors:** Veronika Seblova, Jovana Sadlova, Simon Carpenter, Petr Volf

**Affiliations:** 1 Department of Parasitology, Faculty of Science, Charles University in Prague, Prague 2 128 44, Czech Republic; 2 Institute for Animal Health (IAH), Pirbright, Surrey GU24 0NF, UK

**Keywords:** *Culicoides*, *Phlebotomus*, *Leishmania*

## Abstract

Sand flies remain the only proven vectors of *Leishmania* spp. but recent implementation of PCR techniques has led to increasing speculation about "alternative vectors", including biting midges. Here, we summarize that PCR has considerable limits for studing the role of bloodsucking arthropods in the epidemiology of leishmaniasis. The *Leishmania* life cycle in the sand fly includes a complex series of interactions which are in many cases species-specific, the early phase of the infection is, however, non-specific to sand flies. These facts should be considered in detection of *Leishmania* in ,"alternative" or "new" vectors to avoid mistaken speculation about their vector competence.

## Correspondence

Slama *et al.*[1] recently published a report in *Parasites and Vectors* on the discovery of *Leishmania infantum* DNA in two bloodfed *Culicoides* species, *Culicoides imicola* and *C. circumscriptus*. While this demonstrates that *Culicoides* feed on hosts infected with *L. infantum* in Tunisia, they also extend this to suggesting biological transmission of the pathogen. In doing so, they repeatedly support their speculation by referring to our work, Seblova *et al*. [2] published in the *Journal of Medical Entomology*. Quoting directly, they state that Seblova *et al.*[2] “had proved the susceptibility of *C. nubeculosus* to *L. infantum* infection“. This is unfortunate as a directly opposite conclusion was reached in our paper, we demonstrated that the population of *C. nubeculosus* tested did not support development of *L. infantum* and *L. major* and hence the probability of this species acting as a vector of *Leishmania* species infecting humans was negligable. Therefore, we would like to react and avoid misleading other readers.

The implication of alternative vectors of *Leishmania* parasites, like ticks, fleas and biting midges, in the transmission cycle of *Leishmania* parasites is repeatedly discussed in the literature. The role of the brown dog tick, *Rhiphicephalus sanguineus,* was investigated in relation to the epidemiology of canine leishmaniasis caused by *L. infantum*[3-5] but its significance remains uncertain. Similarly, biting midges of subgenus *Forcipomyia* (*Lasiohelea*) were incriminated as vectors of *L. enrietti* complex causing cutaneous leishmaniasis in red kangoroos [6] but this parasite-vector relationship differed significantly from that studied by Slama *et al.*[1]. Other *Leishmania* were PCR-detected even in non-parasitic insects; the finding of *Leishmania tarentolae* in sarcophagid fly can be explained by licking a wound of an injured gecko [7].

The *Leishmania* life cycle in the vector gut includes several morphological forms and a complex series of interactions which are in many cases species-specific [8]. There are several natural barriers, like proteolytic enzymes, the peritrophic matrix and sand fly immune molecules, all of which require adaptation in the *Leishmania* promastigotes to overcome. As blood digestion proceeds, *Leishmania* needs to bind to midgut epithelium to avoid being excreted with bloodmeal remnants. In natural vectors they then migrate forwards, attach to the stomodeal valve and transform to infective metacyclic forms [8]. The early phase of infection in the vector is, however, non-specific; almost any *Leishmania* survive and divide within the sand fly bloodmeal, even in members of the genus *Sergentomyia*, but they thrive only till defecation [9].

Similarly, in experimentally infected *Culicoides nubeculosus* Seblova *et al.* (2012) demonstrated that *Leishmania major* and *L. infantum* developed only early-stage infections and then they are defecated with bloodmeal remnants. Interestingly, the PCR assay detected parasite DNA post-defecation, until day 7 post-infection, despite the microscopical examination revealing that at this time point there were no living parasites. These observations correspond to the study of Coutinho *et al.*[10] or Paz *et al.*[4] where PCR detection of *Leishmania* DNA was accompanied by microscopical observation. Although the PCR assay detected *Leishmania* DNA, no viable *Leishmania* promastigotes were identified. These results cannot be interpreted simply by lower detection threshold of microscopical techniques. Myskova *et al.*[11] quantified *Leishmania* parasites in experimentally infected sand flies before and after bloodmeal defecation by quantitative PCR and two "traditional" methods, estimation *in situ* and direct counting with the aid of hemocytometer. No significant differences were found between microscopical observation *in situ* and Q-PCR after the bloodmeal was passed. Rather, it is necessary to note that the presence of the amplification products does not imply that target organisms were viable due to the persistance of DNA after cell death. This fact should be considered particularly in PCR detection of parasites in "alternative“ or "new" vectors to avoid mistaken speculation about their vector competence.

We are aware that recent spread of PCR techniques broadly available to laboratories with little experience in vector aspects of parasite life cycles has led to an increasing number of speculations about "new" vectors. As highlighted by Seblova *et al.*[2], PCR has considerable limits for studing the role of bloodsucking arthropods in the epidemiology of leishmaniasis. Microscopical observation of *Leishmania* morphological stages in invertebrates, however, remains the gold standard of detection enabling assessment of localization in the gut at different time points post-bloodmeal. At present, therefore, phlebotominae sand flies should remain the only proven biological vectors of *Leishmania* species infecting humans until convincing evidence is provided to the contrary.

## Competing interest

The authors declare that they have no competing interests.

## Authors’ contributions

VS wrote the initial draft. All authors read and approved the final manuscript.
